# Truth in Science Publishing: A Personal Perspective

**DOI:** 10.1371/journal.pbio.1002547

**Published:** 2016-08-26

**Authors:** Thomas C. Südhof

**Affiliations:** Department of Molecular and Cellular Physiology and Howard Hughes Medical Institute, Stanford University Medical School, Stanford, California, United States of America

## Abstract

Scientists, public servants, and patient advocates alike increasingly question the validity of published scientific results, endangering the public’s acceptance of science. Here, I argue that emerging flaws in the integrity of the peer review system are largely responsible. Distortions in peer review are driven by economic forces and enabled by a lack of accountability of journals, editors, and authors. One approach to restoring trust in the validity of published results may be to establish basic rules that render peer review more transparent, such as publishing the reviews (a practice already embraced by some journals) and monitoring not only the track records of authors but also of editors and journals.

## Abstract 2

In this Perspective, Thomas C. Südhof describes some of the current challenges to the peer review system that have endangered public acceptance of science and discusses possible avenues to addressing these challenges.

As scientists, we are part of society. Thus, it may be expected that questions about reproducibility of published studies, which are, in effect, questions about the truthfulness of these studies, are fair game [1,2]. After all, much science is publicly funded, and the public has a right to ask whether their funds are well spent. What’s more, in a time when many publicly held and expressed opinions are patently false (although it is not always clear whether the people who express these opinions are aware that they are lies), it is important for an engaged citizenry to demand evidence in support of claims. For too long, elected officials in the United States have not been challenged when they claim, for example, that global warming does not exist, that tax cuts will increase tax revenues, and that government-run health insurance is less efficient than privately run health insurance, even though in each case, the evidence indicates the opposite.

But scientists, unlike politicians, are supposed to be guided by facts. Scientists should be insulated from the patent disregard of facts in public discourse. Why, then, are increasing numbers of scientific studies becoming suspect? In my personal view, we as scientists must accept at least part of the blame for failing to ensure that studies report facts and not fantasy. The increasingly severe problem here stems not from outright fraud, which continues to be rare. Instead, the problem is due to biased interpretations of experimental results. These interpretations lead to exaggerated statements of fact (a.k.a. conclusions), which not infrequently are only distantly related to the actual data in a paper.

As a concrete example, the last decade witnessed hundreds of neuroscience papers in which an animal’s behavior is analyzed after defined populations of neurons were excited or inhibited using optogenetic methods. These papers provide valuable information, but commonly conclude that the manipulated neurons physiologically perform the studied behaviors, which change during the manipulations. However, these manipulations induce massive changes in neuronal activity that do not replicate the normal operation of the affected neurons. Moreover, the large activity changes that are induced propagate throughout the brain and thus likely induce myriads of downstream effects. As a result, conclusions from such experiments about the normal functions of the manipulated neurons are difficult to sustain without complementary, independent evidence [3]. Indeed, sometimes ablating the same population of neurons whose optic manipulation produces major effects has no behavioral consequences [4].

Two checkpoints are meant to safeguard scientific truth and to prevent unjustified conclusions: peer review and reproducibility. Peer review examines the validity of the experiments and conclusions as presented, and reproducibility ensures that the experimental results and conclusions can be replicated. Both checkpoints are under threat primarily due to economic factors.

## Compromised Peer Review

Peer review of scientific results occurs at multiple levels, among which the review of scientific manuscripts is arguably the most important because published papers provide the reference point for all other types of peer review (such as selection of grants, promotions, lecture invitations, and prizes). Given the vast amount of data produced, journals serve a valuable role in identifying results and conclusions that merit attention—nobody can possibly read all papers in a field! In recent times, the role of journals in selecting studies has become all-powerful. At some journals, editors who direct the selection process have become akin to high priests and priestesses of science, and here a whiff of ancient Egypt with pharaohs controlling access to wisdom can permeate the review process.

As a result, three problems have emerged in peer review that have corrupted the process, decreasing its value. First, journals and their peer reviewers often have a conflict of interest that is hidden. Journal profits depend on the broadness of the readership and on advertisers, leading to geographical biases (articles from economically important countries are preferred) and content biases (articles on trendy subjects are selected), while reviewers may have other agendas (e.g., supporting friends or holding an economic or professional stake in the results). Sometimes journals and reviewers may not even be aware of the corrupting influence of commercial interests. Attacking this problem will require more than just declaring consulting and ownership relationships. In an ideal world, a journal should not be funded by advertisers or subscribers but by authors’ fees, and reviewers should recuse themselves in cases of commercial and personal conflicts. As argued below, at a minimum, journals should be held accountable not only by their owners for the money they make but also by the public for the value they provide—just as a drug company cannot simply sell any drug but has to show that the drug is safe and effective, a journal should not be allowed to “sell” its products without being accountable for its content.

This brings me to the second problem related to peer review: there is little accountability for journals and reviewers. If a journal repeatedly publishes papers that draw untenable conclusions, eventually the authors of the papers may be blamed, but editors and reviewers who are arguably responsible for gross negligence are not held responsible. There are insufficient checks and balances in the publishing system; when high-ranked journals repeatedly publish papers that are later considered unreliable or even retracted, the journals seem to face no consequences—their premier status remains untouched.

The third peer review problem, finally, is that there is no real competition between journals as the conduit for communicating science. Capitalism thrives and depends on competition. Just like in many other commercial domains nowadays, however, authors have no true choice between journals. The majority of high-profile journals are run by a few companies with significant profits, and it is very hard for newcomers to break into this system. The lack of journal competition means that authors have limited choices in selecting journals with better peer review, decreasing the economic pressure on journals to invest in high-quality peer review.

## Endangered Reproducibility

The other pillar of scientific truth, reproducibility, means that another scientist can repeat an experiment and arrive at the same results or, conversely, show that the results are not reproducible. Just as for peer review, multiple problems increasingly imperil reproducibility. For example, it’s not uncommon for an initial high-profile study to report amazing results with a stunning conclusion. Then, when the experiments are repeated, only trends toward the same conclusion are observed with increasingly smaller effect sizes. This outcome neither contradicts nor confirms the original study but is a dead end, and the original paper is slowly forgotten. As discussed above, the problem is not that the initial paper is fraudulent, but that the results were “tweaked” or selected, or represented a statistical outlier, leading to a misleading conclusion.

A second emerging reproducibility problem is that many experiments are by design impossible to repeat. As formalized by Karl Popper [5], scientific truth requires interpersonal reproducibility. Based on this postulate, any conclusion that cannot be falsified because the underlying experiment cannot be repeated in exactly the same way is not a scientific conclusion. Many current experiments are so complex that differences in outcome can always be attributed to differences in experimental conditions (as is the case for many recent neuroscience studies because of the complexity of the nervous system). If an experiment depends on multiple variables that cannot be reliably held constant, the scientific community should not accept the conclusions from such an experiment as true or false. Such conclusions are simply non-scientific, even if based on an experiment.

A third reproducibility problem is validation of reagents and methods. Too often, papers in premier journals are published without sufficient experimental controls—they take up too much space in precious journal real estate!—or with reagents that have not been vetted after they were acquired. Added to these reproducibility problems is the near impossibility of actually publishing negative results, owing to the reluctance of journals—largely motivated by economic pressures—to devote precious space to such papers, and to the reluctance of authors to acknowledge mistakes.

## Towards More Reliable Results

Thus, we as the scientific community face major problems in ensuring the legitimacy of science. Although correcting these problems will not be trivial, simple steps could increase scientific truthfulness. In my personal view, there is no alternative to journals—we need journals as a filter, now more than ever, and journals need to be economically sustainable. However, given the robust profit margins of many journals, I feel that it is reasonable to insist that scientific journals adhere to a minimum set of rules. For example, reviews should be published, not hidden. Editors should be named as part of the published reviews and should be held accountable if papers fail to meet basic quality and reproducibility standards. Papers should be evaluated historically, not by citations (which can be misleading), but by tracking the follow-up to these papers. At least, the more prominent journals should systematically monitor subsequent work (or lack thereof) emerging from important studies. Submitted papers should be assessed by a checklist that ensures that proper controls and reagent validations are present, and such validations should be required for the supplementary materials. It is amazing how many prominent papers show immunoblots in which the supposed target proteins have the wrong size! Editors need to have the qualifications for judging the overall technical validity of experiments even if they cannot assess specific details (which is the job of the reviewers). Moreover, editors need to have the time to carefully read the papers and to understand the methods and experiments, and they should be paid better for the vast amount of work they are asked to do. Most importantly, as reviewers, we should emphasize less how exciting a result is even though it may not be true and focus more on whether a result is actually solid (i.e., true).

A more demanding but possibly necessary change to ensure scientific truthfulness is to demonstrate immediate reproducibility. A conclusion should not be based on a single type of measurement, but on multiple parallel approaches. Ideally, scientists should recruit other groups to independently reproduce key results. Most pressing, however, in ensuring validity of scientific studies may be what I would call the common sense rule: the more a paper arouses amazement because it appears to contravene common sense and/or because it arrives at conclusions that diametrically differ from previous studies (also referred to as “novelty”), the more evidence should be required. Occasionally, studies that most challenge credulity are published with the least actual experimental support because such studies exude excitement—however, these are the studies that require the most experimental support!

Many of these ideas have been expressed multiple times before. Never, however, has the need for action been more urgent than now, when our entire society is increasingly threatened by untruthfulness, with science being only a tiny part of it. Because the driving factors behind the threats to scientific practice are economic and political, we should speak up and express our concerns. As “voluntary” action seems unlikely, we should demand rules that inject accountability into the system, as capitalism without rules appears to become self-destructive and leads to self-sustaining and self-serving monopolies that impede progress.
